# Heat Shock Factor 1 Prevents Age-Related Hearing Loss by Decreasing Endoplasmic Reticulum Stress

**DOI:** 10.3390/cells10092454

**Published:** 2021-09-17

**Authors:** Yun Yeong Lee, Eun Sol Gil, In Hye Jeong, Hantai Kim, Jeong Hun Jang, Yun-Hoon Choung

**Affiliations:** 1 Department of Otolaryngology, School of Medicine, Ajou University, Suwon 16499, Korea; seven260@naver.com (Y.Y.L.); gilles1996@naver.com (E.S.G.); inhee465@naver.com (I.H.J.); noto.hantai@gmail.com (H.K.); jhj@ajou.ac.kr (J.H.J.); 2 Department of Biomedical Sciences, Graduate School of Medicine, Ajou University, Suwon 16499, Korea

**Keywords:** age-related hearing loss, apoptosis, endoplasmic reticulum stress, heat shock factor 1, heat shock protein

## Abstract

Endoplasmic reticulum (ER) stress is a common stress factor during the aging process. Heat shock factor 1 (HSF1) plays a critical role in ER stress; however, its exact function in age-related hearing loss (ARHL) has not been fully elucidated. The purpose of the present study was to identify the role of HSF1 in ARHL. In this study, we demonstrated that the loss of inner and outer hair cells and their supporting cells was predominant in the high-frequency region (basal turn, 32 kHz) in ARHL cochleae. In the aging cochlea, levels of the ER stress marker proteins p-eIF2α and CHOP increased as HSF1 protein levels decreased. The levels of various heat shock proteins (HSPs) also decreased, including HSP70 and HSP40, which were markedly downregulated, and the expression levels of Bax and cleaved caspase-3 apoptosis-related proteins were increased. However, HSF1 overexpression showed significant hearing protection effects in the high-frequency region (basal turn, 32 kHz) by decreasing CHOP and cleaved caspase-3 and increasing the HSP40 and HSP70 proteins. These findings were confirmed by HSF1 functional studies using an auditory cell model. Therefore, we propose that HSF1 can function as a mediator to prevent ARHL by decreasing ER stress-dependent apoptosis in the aging cochlea.

## 1. Introduction

Age-related hearing loss (ARHL), a progressive form of bilateral hearing loss, is among the most common disorders affecting older people. Exposure to noise, ototoxic drugs, or genetic factors can influence the onset of ARHL [1]. Previous studies have reported that mitochondrial DNA mutation during aging can lead to mitochondrial defects and generate reactive oxygen species (ROS), followed by an increase in apoptosis contributing to cochlear pathology [2,3]. However, the mechanisms underlying these processes remain unclear because insufficient studies have examined the various stress responses causing apoptosis in ARHL.

The heat shock response or endoplasmic reticulum (ER) stress leads to activation of the genes encoding heat shock proteins (HSPs). HSPs function as molecular chaperones for the refolding or degradation of damaged proteins, thereby contributing to intracellular homeostasis. 

The unfolded protein response (UPR) is an intracellular signal transduction pathway that monitors endoplasmic reticulum (ER) homeostasis. Its activation is required to alleviate the effects of ER stress. In mammalian cells, the UPR is controlled by three ER-resident transmembrane proteins, inositol-requiring enyzme-1 (IRE1), PKR-like ER kinase (PERK), and activating transcription factor-6 (ATF6), by which cytoprotective mechanisms are initiated to restore ER functions. However, sustained and unresolved ER stress can eventually trigger cell death by inducing pro-apoptotic proteins such as C/EBP homologous protein (CHOP), primarily through the PERK/eIF2α/ATF4 pathway [4,5,6].

The cross-link between hearing loss and endoplasmic reticulum (ER) stress was recently demonstrated in animal and cell experiments that showed that ER stress is involved in ototoxic drug effects, prolonged noise exposure, and ARHL [7,8,9]. Notably, immunoglobulin heavy chain-protein (BiP), also known as glucose-regulated protein 78 (GRP78), expression was found to be decreased, whereas CHOP expression was increased, in the cochleae of aged mice, and the cleavage of caspase-3 and -9, but not -12, was consistently increased in aged cochleae [10], suggesting impairment of the unfolded protein response pathway and activation of the cell death pathway.

Accordingly, HSF1 has been implicated in several fundamental biological processes independent of heat shock responses, including metabolism, gametogenesis, development, and aging, as well as in various pathologies, especially neurodegenerative disorders and cancer [11,12,13,14]. Activated HSF1 migrates into the nucleus, where it promotes HSP gene transcription [15,16] and induces chaperone gene expression by binding to DNA sequence motifs known as heat shock elements (HSEs) [17,18]. HSF1 controls chaperone-mediated protein folding by transcriptional upregulation of HSPs such as *Hspa1a, Hspa8, Hspa6, Dnajb1, St13,* and *Fkbp4*, suggesting diverse roles for HSF1 that extend far beyond heat shock responses [19].

HSF1 overexpression in mammalian cells has been shown to lead to HSP upregulation and increased cellular protection against heat shock and ischemia stress. HSF1 has been found in the outer (OHCs) and inner hair cells (IHCs) of the organ of Corti (OC) in the stria vascularis and in spiral ganglion cells of the modiolus in normal rat and mouse cochleae [20]. These previous studies demonstrated that heat shock preconditions the cochlea against noise trauma [21], resulting in HSF1 activation in rodent cochleae [20].

A recent study showed that the loss of hair cells was a more significant factor of ARHL than stria degeneration, with higher cell loss levels observed in human samples than in several animal models. Apoptosis is a major cause of cell death; however, the specific mechanisms involved in the various stress responses that induce apoptosis remain poorly understood due to their complexity. In particular, the function of HSF1 in ER stress-mediated UPR branch apoptosis pathways in the cochlea has not been investigated. Therefore, we analyzed HSF1 expression in young and aged cochlear tissues and investigated the role of ER stress-mediated UPR branch apoptosis pathways on hearing through regulation of HSF1 expression and the mechanisms involved in the auditory cell model.

## 2. Materials and Methods

### 2.1. Animals

Age-matched male C57BL/6J mice (7 weeks of age) were obtained from Dae Han Bio Link Co., Ltd. (Chungbuk, Korea). All procedures involving animals were approved by the Institutional Animal Care and Use Committee of Ajou University Graduate School of Medicine, Suwon, Republic of Korea (2017-0004). The animals were maintained under standard animal-house conditions. 

### 2.2. Experimental Procedures

To determine HSF1 function, 9-month-old mice (C57BL/6J) were divided into vector (n = 8) and HSF1 (n = 10) groups. We overexpressed mGFP-tagged *vector* control or *hsf1* Lenti-viral infection (about 10^7^ TU/mL) to the cochlea via intratympanic injection (ITI). We established ARHL in vector or HSF1 mice at 12 months. Auditory thresholds were measured in terms of auditory brainstem response (ABR) (8, 16, or 32 kHz) before and after a threshold age of 12 months. After experiments, the mice were sacrificed and analyzed by histology with hematoxylin and eosin (H & E) staining, immunohistochemistry, immunoblotting, and quantitative polymerase chain reaction (qPCR). 

### 2.3. Auditory Brainstem Response Analysis

The ABR threshold was measured as previously described [22]. Briefly, mice were anesthetized with an intraperitoneal injection of ketamine (125 mg/kg) and xylazine (2.5 mg/kg), and then placed in a soundproof chamber to exclude outside noise. The ABR was defined as the lowest stimulus level at which a waveform was visible in the evoked trace using the TDT II System and BioSig software (Tucker Davis Technologies, MathWorks, FL, USA). Frequency-specific ABRs in response to tone-burst stimuli were recorded at 8, 16, and 32 kHz.

### 2.4. IHC, OHC, and SP Counts

Morphometric assessments of IHCs, OHCs, and their supporting cells (SPs) were performed for apical to basal cochlear turns on H & E-stained sections. The cochlear specimens were observed and photographed using bright-field microscopy (Olympus BX-60, Shibuya-ku, Tokyo, Japan) and digital images were recorded. IHCs, OHCs, and SPs were evaluated as the number of cell nuclei per captured image (n = 8 for each group). 

### 2.5. House Ear Institute-Organ of Corti 1 (HEI-OC1) Cell Culture

HEI-OC1 cells were cultured in DMEM (Gibco-BRL, Grand Island, NY, USA) containing 10% FBS (Gibco-BRL, Grand Island, NY, USA) under non-permissive conditions (37 °C, 5% CO_2_) without antibiotics. In heat-shock experiments, we exposed cells to 42 °C and 5% CO_2_ at specific time points.

### 2.6. Immunoblotting

Cochlea tissue extracts were prepared using Polytron homogenization followed by extraction with radioimmunoprecipitation assay (RIPA) buffer containing 50 mM Tris/HCl (pH 7.5), 150 mM NaCl, 1.0% Nonidet P40, 0.1% sodium dodecyl sulfate (SDS), 0.5% deoxycholic acid, 1.0 μg/mL leupeptin, 100 μg/mL phenylmethylsulfonyl fluoride (PMSF), 1.0 mM Na_3_VO_4_, and 1.0 mM NaF. Then, the cells were solubilized in RIPA buffer and insoluble proteins were eliminated by centrifugation at 12,000× *g* for 10 min at 4 °C, and a 40-μg protein sample was separated using sodium dodecyl sulfate polyacrylamide gel electrophoresis (SDS-PAGE). After electrophoresis, the proteins were transferred to polyvinylidene fluoride (PVDF) membranes (Millipore, Bedford, MA, USA) and blocked with 5% non-fat dried milk in 0.1 M phosphate-buffered saline (PBS, pH 7.4) containing 0.05% Tween 20 (PBST) for 1 h. The membranes were incubated with the appropriate antibodies overnight at 4 °C. The membranes were washed three times with PBST and incubated with the appropriate horseradish-peroxidase-conjugated secondary antibodies for 1 h. After washing, an enhanced chemiluminescence (ECL; Amersham Biosciences, Little Chalfont, UK) kit was used to evaluate protein levels. Band intensity was analyzed using ImageJ software (available for free download at https://imagej.nih.gov/ij/).

### 2.7. Immunohistochemistry

The cochlea was dissected, fixed with 4% paraformaldehyde, and decalcified in Calci-Clear Rapid Decalcifying Solution (National Diagnostics, Atlanta, GA, USA) for 4 days. Decalcified cochleae were embedded in paraffin for immunofluorescent histological analysis, paraffin-embedded sections (6 μm thick) were dried for 30 min on a slide warmer at 60 °C, deparaffinized, rehydrated in a graded alcohol series (100%, 90%, 80%, and 70%) and then stained with H&E. To retrieve antigens for immunohistochemistry, the sections were placed in sodium citrate buffer (10 mM sodium citrate, 0.05% Tween 20, pH 6.0), heated to 95 °C in plastic Coplin jars for 30 min and cooled at room temperature (RT) for 30 min. Next, endogenous peroxidase activity was blocked in 3% H_2_O_2_ (Sigma-Aldrich, St. Louis, MO, USA) at RT for 10 min. The slides were incubated with 3% bovine serum albumin (BSA; GenDEPOT, Barker, TX, USA) and 0.05% Triton X-100 in 0.1 M PBS. Then, sections were incubated overnight with primary antibodies diluted in blocking buffer with gentle agitation at 4 °C. After three washes with 0.1 M PBS, the sections were incubated at RT for 1 h with the appropriate secondary antibodies. The sections were washed three times in 0.1 M PBS and counterstained with 4′,6-diamidino-2-phenylindole (DAPI). The primary antibody was replaced with non-immune serum for negative controls. The sections were visualized under a confocal microscope (Carl Zeiss Microscopy, Jena, Germany).

### 2.8. Cell Viability Assay

The water-soluble tetrazolium salt (WST-1) assay was used to analyze cell viability (Cayman Chemical, Ann Arbor, MI, USA). HEI-OC1 cells were cultured in 96-well plates at a density of 1 × 10^4^ cells/well for 24 h. Then, WST-1 was added to all wells and the plate was incubated for 2 h according to the manufacturer’s protocol. The absorbance at 450 nm was measured. All assays were performed at least in triplicate using an iMark Microplate Reader (Bio-Rad, Hercules, CA, USA).

### 2.9. Doxorubicin (DOXO) Treatment

HEI-OC1 cells were cultured in six-well plates at a density of 8 × 10^4^ cells/well with or without a coverslip and exposed to DOXO (100 ng/mL) for 5 days. The cells were maintained in complete medium without antibiotics. DOXO-induced cellular senescence was confirmed by assaying the expression of p53 and p21^WAF1/Cip^, senescence-associated-β-gal (SA-β-gal), and BrdU; the cell cycle was analyzed at the indicated times.

### 2.10. Senescence-Associated Β-Gal Assay

HEI-OC1 cells were fixed in 4% paraformaldehyde in 0.1 M PBS for 5 min and incubated at 37 °C overnight in staining solution (1.0 mg X-Gal/mL dimethylformamide, 40 mM citric acid/sodium phosphate, pH 6.0, 5 mM potassium ferrocyanide, 5 mM potassium ferricyanide, 150 mM NaCl, and 2 mM MgCl_2_). Flow cytometric analysis of SA-β-gal was performed using a Quantitative Cellular Senescence Assay Kit (Cell Biolabs, San Diego, CA, USA). Briefly, cells were treated with pretreatment solution at 37 °C for 2 h. Then, the cells were incubated in SA-β-gal substrate for 4 h. Finally, the stained cells were washed with 0.1 M PBS and harvested by trypsinization.

### 2.11. Brdu Incorporation Assay

Vehicle- or DOXO-treated HEI-OC1 cells were labeled with 10 μM BrdU (Sigma, ST Louis, MO, USA) for 4 h, fixed in 4% paraformaldehyde, and incubated in 2 M HCl for 30 min. The pH was increased by adding 0.1 M sodium borate (pH 8.5) for 2 min. Next, the samples were incubated with 3% BSA in 0.05% Triton X-100 for 2 h. Anti-BrdU primary antibody was applied at 4 °C overnight, followed by the secondary antibody for 2 h. We performed counterstaining with DAPI. The positive cells were visualized under a confocal microscope.

### 2.12. Cell Cycle Analysis

Cells were treated with vehicle or DOXO for 5 days, and then trypsinized, fixed, and re-suspended in propidium iodide solution. DNA analysis was performed by flow cytometry (BD Biosciences, Franklin Lakes, NJ, USA).

### 2.13. Real-Time PCR

Total RNA was extracted using RNAiso Plus (TaKaRa, Shiga, Japan) and cDNA was synthesized from 1.0 μg RNA using a reverse transcription kit (Invitrogen, Carlsbad, CA, USA) according to the manufacturers’ instructions. cDNA was amplified in a CFX96 Real-Time PCR Cycler (Bio-Rad) using appropriate primers and SYBR Green PCR Master Mix (Applied Biosystems, Foster City, CA, USA) with activation at 95 °C for 15 min, followed by 40 cycles of 95 °C for 20 s and 60 °C for 30 s. Supplementary Table S1 lists the primers used. The expression of the genes of interest is expressed as the fold change over the control.

### 2.14. siRNA Transfection

Control siRNAs (sc-37007) and siRNA against HSF1 (sc-35612) were purchased from Santa Cruz Biotechnology. The cells were transfected with siRNAs and Lipofectamine RNAiMAX Transfection Reagent (Invitrogen) for 48 h, followed by exposure to heat shock at the indicated time points. The cells were then subjected to WST-1 or immunoblot analysis.

### 2.15. HSF1 Viral Production and Titration

We used the Lenti ORF clone of *Hsf1* (mGFP-tagged), Mouse heat shock factor 1 (Hsf1) (MR208087L2; OriGene Technologies Inc., MD, USA). Empty vectors (pLenti-C-mGFP) were prepared by sub-cloning. Briefly, the Lenti-vpak packaging kit was used to package plasmids and transfection reagent (TR30037; OriGene Technologies Inc.) into HEK293T cells to produce lentiviral particles according to the manufacturer’s protocol. The supernatants were harvested 24 and 48 h after transfection, filtered, and added to recipient cell lines with 8 μg/mL hexadimethrine bromide (Sigma-Aldrich). The virus titer was calculated as follows: virus titer (TU/mL) = (average fluorescence per well × cell number)/volume of virus added [23]. Viral particles transduced in vivo or in vitro about 10^7^ TU/mL were used. HSF1 protein levels were quantified by qPCR or western blotting.

### 2.16. Antibodies and Reagents

Antibodies against anti-HSF1 (12972S), HSP90 (4877T), HSP70 (4872T), HSP60, (12165T), HSP40 (4871T), Cleaved caspase-3 (9661S), CHOP (2895S), p-eIF2α (immunoblot; 9721S, immunofluorescence; 3398), Bax (14796S), Bcl-2 (3498S), p53^ser15^ (12571S), p53 (2524S), p21^WAF1/Cip^ (64016S), and β-Actin (3700) were obtained from Cell Signaling Technology. Antibody against anti-MYO7A (sc-25834) was obtained from Santa Cruz Biotechnology.

### 2.17. Statistical Analyses

Data are expressed as means ± standard deviation (SD) or standard error of the mean (SEM) of at least three independent experiments. Differences among means were assessed using Student’s *t* test for two groups and one-way analysis of variance (ANOVA) for multiple groups followed by Tukey’s honest significant difference (HSD) test using the SPSS software (version 23.0, IBM Corporation, Armonk, NY, USA). Significance was evaluated at a level of *p* < 0.05.

## 3. Results

### 3.1. Auditory Hair Cell Loss in Aged Cochlea Was Accompanied by a Decrease in HSF1 Expression and Increased ER Stress and Apoptosis

**Figure 1:**
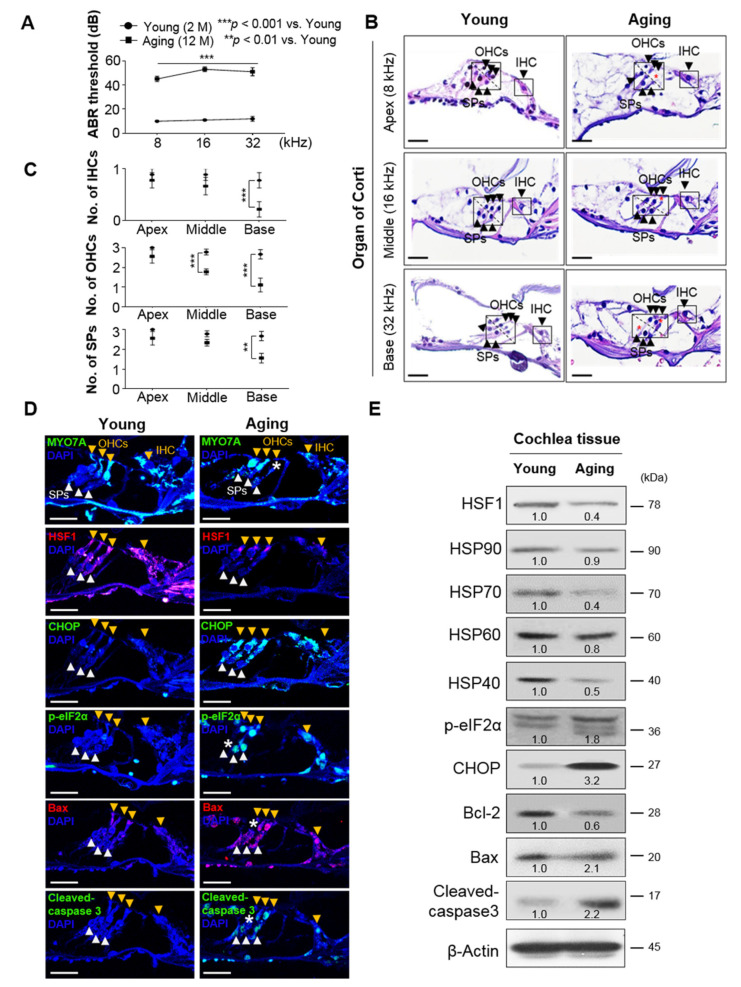
Cochlea of mice with age-related hearing loss (ARHL) exhibit reduced HSF1 and heat shock protein (HSP) expression, and increased expression of endoplasmic reticulum (ER) stress and intrinsic apoptosis markers. (**A**) The auditory brainstem response (ABR) threshold was significantly higher at 8, 16, and 32 kHz in the aging group (n = 12) than in the control group (n = 12). Data are means ± standard error of the mean (SEM). (**B**) Hematoxylin and eosin (H & E)-stained sections from the middle and base turns of the cochlea. Black darts indicate inner (IHCs) and outer hair cells (OHCs) and their supporting cells (SPs) within boxes. Red asterisks indicate cell loss. Scale bars, 30 μm. (**C**) IHC, OHC, and SP counts in young and aging mice from the apex to the basal turn of the cochlea, measured on histological sections. Data are means ± SEM of eight independent sections. (**D**) Sections were immunolabeled with anti-MYO7A, anti-HSF1, anti-CHOP, anti-p-eIF2α and anti-Bax antibodies to evaluate the relationship between ER stress and apoptosis with HSF1 expression levels. DAPI was used as a counterstain. Arrow heads indicate IHCs and OHCs (yellow) and SPs (white). Cell loss is indicated by white asterisks. Scale bars, 20 μm; DAPI was used as a counterstain. Darts indicate IHCs and OHCs (yellow) and SPs. Cell loss is indicated by white asterisks. Scale bars, 20 μm. (**E**) Protein expression was measured in cochlea extracts from the young and aging groups. Relative band intensities are presented as means of the average of at least three independent experiments. Densitometry experiments were performed using ImageJ software (Supplementary Figure S5). All experiments were performed with at least three replications for each condition and repeated at least twice ** *p* < 0.01; *** *p* < 0.001 (one-way analysis of variance (ANOVA), followed by Tukey’s honest significant difference (HSD) test).

The ABR threshold was significantly higher in aging mice (12 months) compared to in younger mice (8 weeks), resulting in ARHL at all frequencies (8 kHz, 35 dB ± 4.4; 16 kHz, 42 dB ± 4.1; 32 kHz, 39 dB ± 6.6, respectively) (Figure 1A). Cochlea histology and quantitative analyses showed significant decreases in IHCs in the basal turn area, OHCs in the middle and base turns, and SPs in the basal turn area in aging mice (Figure 1B,C). Compared to the younger group, the aging group had significantly fewer spiral ganglion neurons (SGNs) (Supplementary Figure S1A,B). This loss of hair cells, SPs, and SGNs is a representative characteristic of age-related hearing loss and is consistent with the findings of previous studies [24,25,26].

Apoptosis is caused by a variety of stressors, especially ER stress [27]. In IHCs, OHCs, and SPs of the OC, the expression of the ER stress markers p-eIF2α and CHOP, as well as the apoptosis markers Bax and cleaved-caspase 3, were significantly higher in the aging group compared to the control group, indicating high ER stress and apoptosis (Figure 1D). Immunofluorescence confirmed significantly lower HSF1 expression in the aging group (Figure 1D). Levels of the ER stress markers p-eIF2α and CHOP and apoptosis markers Bax and cleaved caspase-3 increased and those of HSPs decreased in the aging group, with significant decreases in HSP70 and HSP40 (Figure 1E). Based on these results, we inferred that HSF1, HSP, and Bcl-2 expression are inhibited in aged cochleae, followed by intrinsic apoptosis mediated by CHOP and Bax, resulting in cell loss.

### 3.2. During Cellular Senescence, the Expression of HSF1 and HSP Decreases, While the Expression of ER Stress and Apoptosis Markers Increases

**Figure 2:**
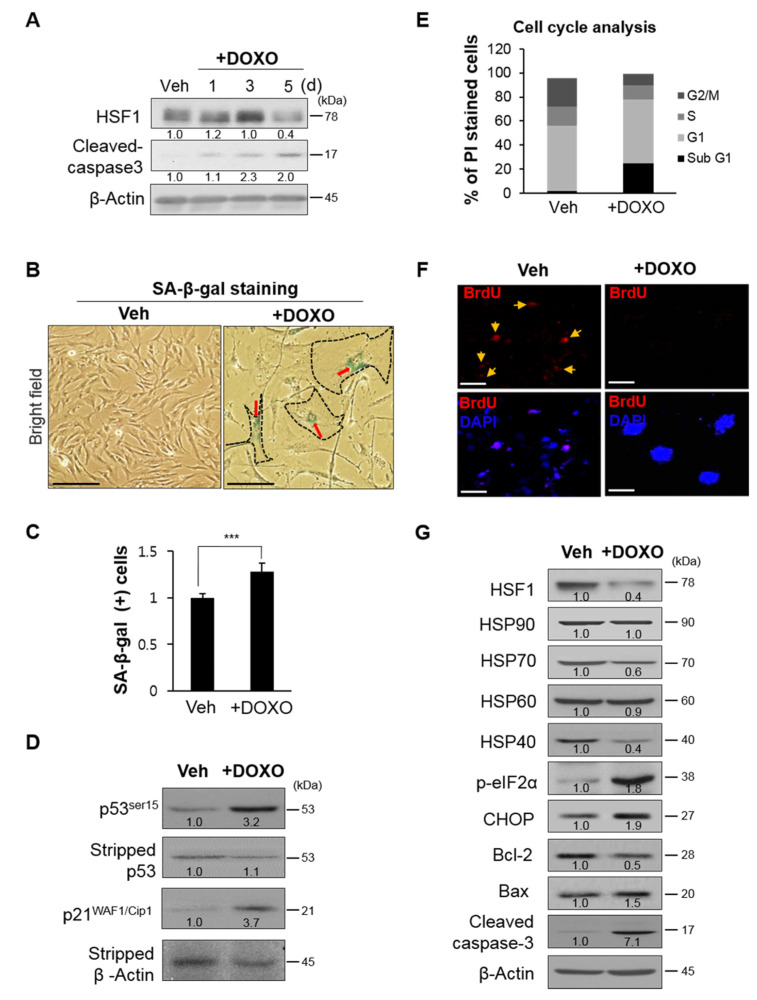
Decreases in HSF1 and HSP expression were accompanied by increased ER stress and apoptosis markers in premature senescence. (**A**) HEI-OC1 cells were treated with doxorubicin (DOXO; 100 ng/mL) and maintained for 5 days. HSF1 and cleaved caspase-3 expression levels were assayed by immunoblotting at the indicated times. β-actin was used as a loading control. (**B**) Senescent cells treated with DOXO for 5 days were stained with SA-β-gal. Dimethylsulfoxide (DMSO)-vehicle-treated cells were used as a control. Black dot line and red arrows indicate SA-β-gal (+) cells. Scale bars, 100 μm. (**C**) Quantification of SA-β-gal (+) cells was achieved by fluorescence-activated cell sorting (FACS). Data are means ± SD. (**D**) Immunoblot analysis shows induction of p53 and p21^WAF1/Cip1^ expression. β-actin was used as a loading control. (**E**) The cell cycle was analyzed in vehicle- and DOXO-treated cells using flow cytometry. (**F**) BrdU incorporation in HEI-OC1 cells after vehicle or DOXO treatment for 5 days. Cells were immunolabeled with anti-BrdU antibodies to evaluate BrdU incorporation into the nucleus. Nuclei were stained with DAPI. Yellow arrows indicate BrdU (+) cells. Scale bars, 20 μm. (**G**) HSF1, heat shock protein (HSP, i.e., HSP90, HSP70, HSP60, and HSP40), ER stress (p-eIF2α and CHOP), and anti- (Bcl-2) and pro-apoptosis (Bax and cleaved caspase-3) markers were analyzed by immunoblotting in cells treated with vehicle or DOXO for 5 days. β-actin was used as a loading control. Relative band intensities are presented as means of the average of at least three independent experiments. Densitometry experiments were performed using ImageJ software (Supplementary Figure S5). All experiments were performed with at least three replications for each condition and repeated at least twice. *** *p* < 0.001 (Student’s *t* test).

Senescence is considered to be an irreversible process of growth arrest occurring in response to cellular aging and various types of cellular stimulation causing DNA damage [28]. This form of senescence is also known as stress-induced premature senescence (SIPS). The cell cycle regulator p53-p21^WAF1/Cip1^ is induced by DNA damage and proliferative arrest, and is a main driver of apoptosis [29,30]. In our in vitro mechanism study, we established a SIPS auditory hair cell model as described in our previous study [31], and made observations for 5 days after treatment with a low-dose DOXO (100 ng/mL), a DNA damage reagent. We found that C-cas3 expression increased as HSF1 decreased after 3 days, which was consistent with our in vivo results (Figure 2A). After 5 days, SIPS features were increasingly prevalent, such as enlarged cell morphology and SA-β-gal staining in positive cells (Figure 2B,C), cell cycle suppression (Figure 2E), decreased BrdU incorporation, and increased nucleus size (Figure 2F). Increased cell death rates and decreased S and G2/M cell cycle transitions were confirmed (Figure 2E). Thus, SIPS cells were established through DOXO administration, confirmed by the increase in p53 and p21^WAF1/Cip1^, which are cell cycle inhibitors (Figure 2D). SIPS cells showed decreased expression of HSF1 and HSPs, with significant decreases in HSP70 and HSP40 expression, whereas the expression of proteins related to ER stress and apoptosis increased (Figure 2G). These results were consistent with our in vivo results, confirming that ER stress-mediated UPR persists due to active p-eIF2α and CHOP, as well as reduced HSF1 and HSP expression, in aged auditory hair cells, which is followed by cell death caused by apoptosis.

### 3.3. HSF1 Overexpression Increased Cell Viability by Inhibiting ER Stress and Apoptosis via HSP Expression Regulation

**Figure 3:**
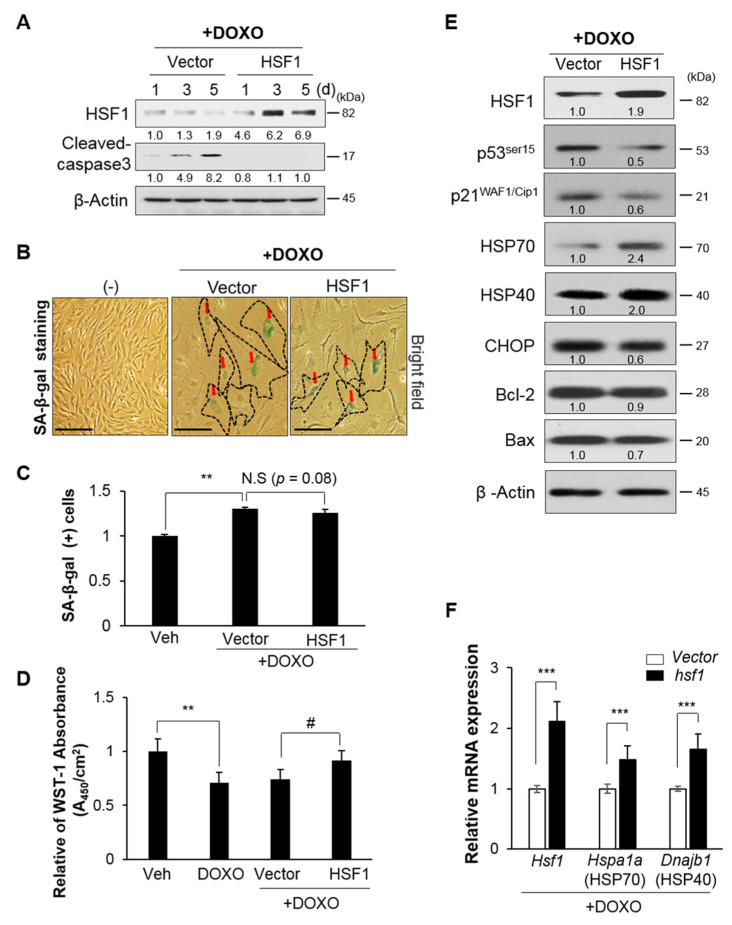
HSF1 overexpression inhibited ER stress-mediated UPR branch apoptosis pathways. (**A**) *hsf1* or *vector* Lenti-viral particles (10^7^ TU/mL) transduced to HEI-OC1 cells were treated with vehicle or DOXO and incubated for 5 days. HSF1 and cleaved caspase-3 expression levels were assayed by immunoblotting at the indicated times. β-actin was used as a loading control. (**B**) Cells were stained with SA-β-gal. Black dot line and red arrows indicate SA-β-gal (+) cells. Scale bars, 100 μm. (**C**) SA-β-gal (+) cells were quantified by flow cytometry. Data are means ± SD. (**D**) Cell viability was determined using a WST-1 assay in viral transduced cells treated with vehicle and DOXO with or without *vector* and *hsf1*. Data are means ± SD. (**E**) HSF1, HSP (HSP70 and HSP40), ER stress (CHOP) and anti- (Bcl-2) and pro-apoptosis (Bax) markers were analyzed by immunoblotting in viral transduced cells treated with DOXO with or without *vector* and *hsf1*. β-actin was used as a loading control. (**F**) *Hsf1*, *Hspa1a* (HSP70), and *Dnajb1* (HSP40) expression levels were measured by quantitative polymerase chain reaction (qPCR). *Ribosomal RNA (18S)* was used as a control. Cells were treated with vehicle or DOXO for 5 days, as shown in figure (**B**–**F**). Data are means ± SD. Relative band intensities are presented as means of the average of at least three independent experiments. Densitometry experiments were performed using ImageJ software (Supplementary Figure S5). All experiments were performed with at least three replications for each condition and repeated at least twice. ^#^ *p* < 0.05, ** *p* < 0.01, *** *p* < 0.001 (Student’s *t* test or one-way ANOVA, followed by Tukey’s HSD test.

These results indicate that the decreased expression of HSF1 and HSP during aging is closely related to the increase in ER stress and apoptosis. Therefore, we investigated the effects of HSF1 protein overexpression on cellular senescence and death by examining HEI-OC1 cells transfected with either *vector* control or *hsf1* virus at days 1, 3, and 5 after treatment with DOXO. We observed that protein expression was maintained for 3–5 days in the HSF1 overexpression condition, unlike in the vector control. Cleaved caspase-3 expression was decreased by HSF1 overexpression (Figure 3A); however, no decrease in SA-β-gal (+) cells was observed (Figure 3B,C), and WST analysis showed increased cell viability (Figure 3D), likely to be due to an increase in HSP (HSP70 and HSP40) expression and decreases in the cell cycle inhibitors p53 and p21^WAF1/Cip1^ and ER stress markers (Figure 3E,F). Together, these results show that increased HSF1 expression at the beginning of the aging process can control apoptosis and cell cycle inhibition, while relieving ER stress via increased HSP protein expression.

### 3.4. Cell Viability and ER Stress Were Regulated by HSF1 Protein Expression under Heat Shock Stress

**Figure 4:**
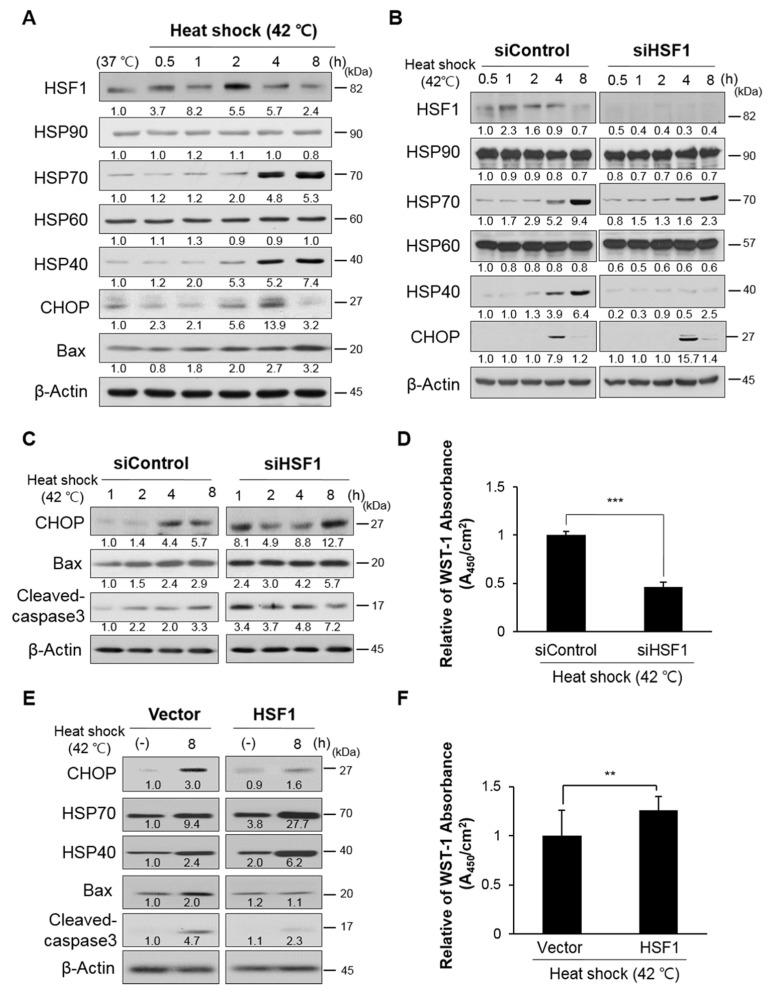
HSF1 expression regulated cell viability via ER stress-mediated UPR branch apoptosis in auditory hair cells. (**A**) HEI-OC1 cells were exposed to heat shock (42°C) at the indicated time points. HSF1, HSP (HSP90, HSP70, HSP60, and HSP40), ER stress (CHOP), and pro-apoptosis (Bax) markers were analyzed by immunoblotting. Cells incubated at 37 °C were used as a normal control. β-actin was used as a loading control. To analyze the effects of HSF1 depletion on heat shock stress, cells were transfected with siControl or siHSF1 for 1 day, and then exposed to heat shock at the indicated time points. The expression levels of (**B**) HSF1, HSPs, and (**C**) ER stress- and apoptosis-related proteins were analyzed by immunoblotting. β-actin was used as a loading control. (**D**) After 24 h of siRNA transduction, the cells were exposed to 42 °C for 8 h, and then incubated with WST-1 at 37 °C for 24 h. Data are means ± SD. (**E**) To analyze the effects of HSF1 transient overexpression on heat shock stress, hsf1 or vector Lenti-viral particles were transduced into HEI-OC1 cells and incubated for 3 days. Following HSF1 overexpression, cells were exposed to heat shock for 8 h. ER stress- and apoptosis-related proteins were analyzed by immunoblotting and (**F**) cell viability was determined after 1 day using a WST-1 assay. β-actin was used as a loading control. Data are means ± SD. Relative band intensities are presented as means of the average of at least three independent experiments. Densitometry experiments were performed using ImageJ software (Supplementary Figure S6). All experiments were performed with at least three replications for each condition and repeated at least twice. ** *p* < 0.01, *** *p* < 0.001 (Student’s *t* test).

ER stress increases HSF1 activity and HSP protein expression following heat shock stress [32]. In this study, cells exposed to heat stress (42 °C) exhibited ER stress; HSF1 activity (nuclear localization and phosphorylation), which increased from 0.5 to 2 h, and then decreased (Supplementary Figure S3A,B). HSP70 and HSP40 were highly expressed at 4 h after heat shock. The expression of ER stress markers was relatively high at 2 and 4 h after heat shock, after which expression of the cell death marker Bax increased at 8 h (Figure 4A,B and Figure S4). Next, we suppressed HSF1 expression using siRNA, inducing ER stress; HSP70 and HSP40 expression levels were significantly reduced compared to siControl (Figure 4B), and apoptosis occurred via Bax and cleaved caspase-3 due to excessive ER stress, accelerating cell death (Figure 4C,D). These results were found to be reversible through HSF1 overexpression (Figure 4E,F), confirming that excessive ER stress in auditory hair cells causes cell death through the UPR branch intrinsic apoptosis pathway. Together, these results suggest that ER stress is involved in apoptosis during auditory hair cell death, and that HSF1 plays an important role in regulating this mechanism.

### 3.5. HSF1 Overexpression Inhibited ER Stress-Mediated UPR Branch Apoptosis in the OC of Aging Mice, Suppressing High-Frequency Hearing Loss

**Figure 5:**
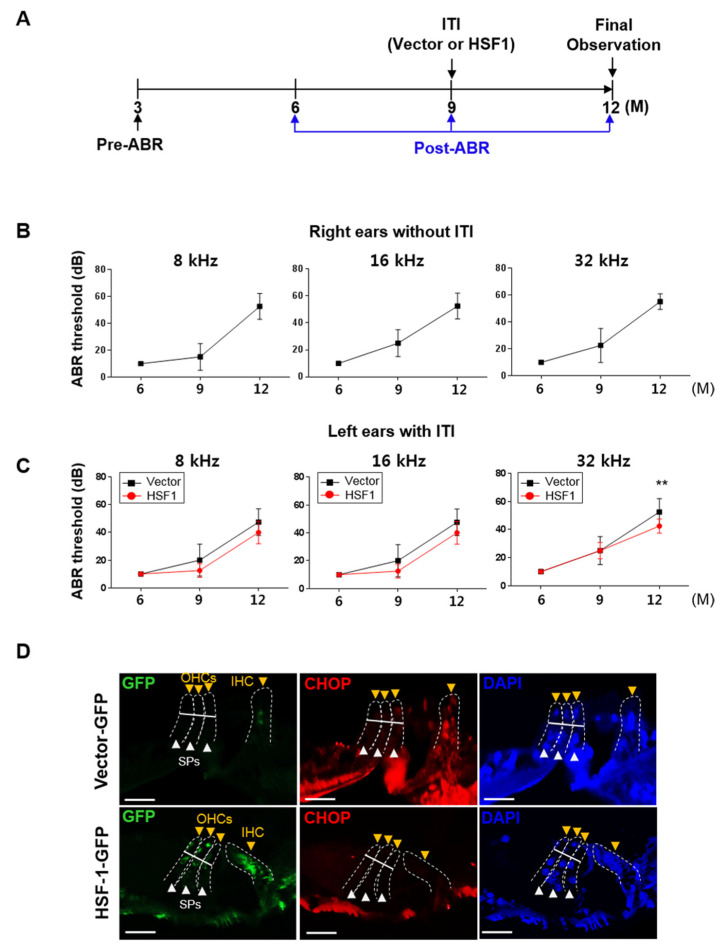
HSF1 overexpression prevented hearing loss at the 32 kHz base turn via decreased ER stress-induced CHOP expression in ARHL mice. (**A**) Normal hearing (pre-ABR) was evaluated in 3-month-old mice (n = 20). Post-ABR was determined from 6 to 12 months. After 9 months, mice were divided into Lenti-viral vector (n = 8) and HSF1 groups (n = 10), with two mice excluded for abnormal hearing, according to the average ABR threshold (≥20 dB). Overexpression of *vector* or *hsf1* (about 10^7^ TU/mL) to the cochlea of the left ear was achieved by intratympanic injection (ITI). Right ears were used as normal controls (without ITI). At 12 months, the final observations were conducted; the mice were sacrificed, and biochemistry analysis was performed. (**B**) ABR thresholds at 8, 16, and 32 kHz in age-dependent normal control right ears (without ITI). Data are means ± SEM of the normal control group (vector and HSF1, all right ears; n = 18). (**C**) The ABR thresholds at 8, 16, and 32 kHz for age-dependent overexpression of vector or HSF1 in right ears (with ITI). Data are means ± SEM of vector (n = 8) and HSF1 (n = 10) left ear groups. (**D**) The high frequency regions of the cochlear sections were subjected to immunohistochemical analyses. The sections were immunolabeled with anti-CHOP antibody to evaluate ER stress compared to mGFP-tagged vector or HSF1 expression. DAPI was used as a counterstain. Arrow heads indicate IHCs and OHCs (yellow) and SPs (white). Scale bars, 20 μm. ** *p* < 0.01 (one-way ANOVA, followed by Tukey’s HSD test).

To investigate the effect of HSF1 on ARHL, we measured ABR in mice for 6, 9, and 12 months (Figure 5A). Based on our preliminary data, ARHL was induced at 9 months (>30 dB, data not shown). Therefore, at 9 months, we introduced *hsf1* or *vector* control lent viral particles into the left ear via ITI, with the right ear used as a normal control. Hearing was compared between the left and right ear during the last 12 months of the experiment. In the right ear (without ITI), the ABR threshold increased after 9 months at 8, 16, and 32 kHz, causing ARHL at 12 months (>40 dB at all frequencies) (Figure 5B). The left ear treated with vector showed similar hearing values to the untreated ear. Hearing in the HSF1-treated ear was not significantly different at 8 or 16 kHz at 9 and 12 months, but the ABR threshold tended to be lower than that of the vector-treated ear. At 32 kHz, the ABR threshold was significantly lower at 12 months (HSF1, 42.5 dB ± 5 vs. vector, 52.5 dB ± 9.5, *p* = 0.016) (Figure 5C). HSF1 expression was higher in IHCs, OHCs, and SPs in tissue sections overexpressed in HSF1 compared to vector control sections, as indicated by green fluorescence protein (GFP); ER stress was significantly lower in the serial section than in the vector control, as indicated by CHOP expression. *Hsf1* (HSF1), *Hspa1a* (HSP70), and *Dnajb1* (HSP40) mRNA and protein expression were higher and cleaved caspase-3 expression was lower in the HSF1 overexpression group (Supplementary Figure S4C,D). Therefore, HSF1 overexpression controlled ARHL by inhibiting ER stress and apoptosis. 

**Figure 6:**
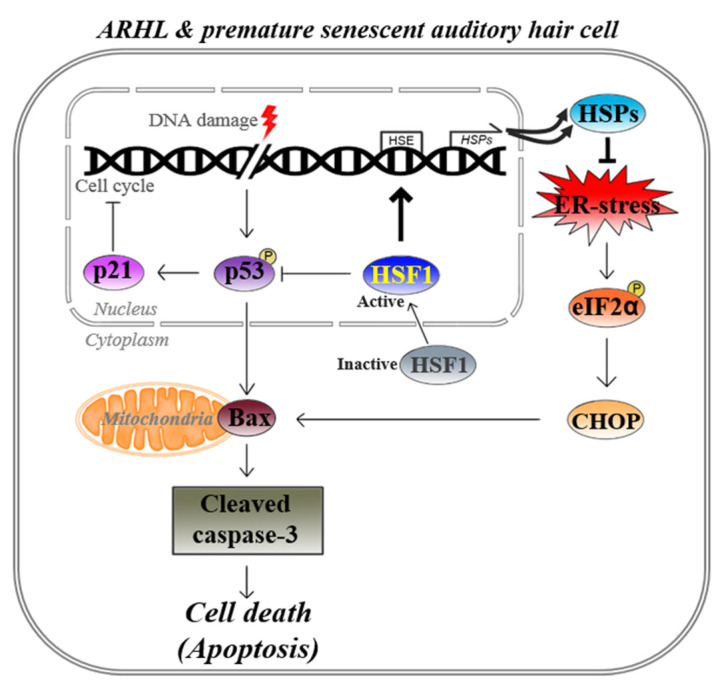
Summary diagram. HSF1 overexpression relieved ER stress through increases in HSP70 and HSP40 gene and protein expression. Reductions in p-eIF2α, CHOP, and p53-p21^WAF1/Cip1^ suppressed Bax–caspase 3-dependent apoptosis, suggesting a mechanism for preserving hearing in the high-frequency regions of ARHL mouse cochleae.

Finally, our in vivo results showed that decreased HSF1 and HSP expression during the aging process caused hearing loss due to a loss of auditory hair cells caused by ER stress-mediated apoptosis. In an in vitro model, HSF1 overexpression inhibited apoptosis by reducing the cell cycle inhibitors p53 and p21^WAF1/Cip1^ and, therefore, ER stress and apoptosis. Thus, HSF1 overexpression increased the expression of HSP70 and HSP40 to relieve ER stress (indicated by p-eIF2α and CHOP) and inhibit auditory hair cell death by reducing apoptosis (indicated by Bax and cleaved caspase-3) to preserve hearing (Figure 6).

## 4. Discussion

The main mechanism of ototoxic drug-induced hearing loss is hair cell loss [33], and the loss of hairs, SGNs, and vascular striated cells is involved in ARHL [34]. The results of this study demonstrated that 8-, 16-, and 32-kHz ABR thresholds were significantly higher in older mice with ARHL than in younger control mice (Figure 1A, upper panel). However, there were significantly fewer IHCs, OHCs, and SPs in the OC of the basal turn (32 kHz), and OHCs and SPs in the middle turn (16 kHz) in the ARHL group than in the younger group. Notably, no significant cell loss was observed in the apex (8 kHz). (Figure 1A, lower panel). This is an important result because hearing loss occurs primarily in high-frequency areas [34,35]. OHCs are more sensitive than IHCs to ototoxic drugs, such as cisplatin, and are therefore more easily damaged. In this study, we observed greater damage to OHCs than to IHCs [36]. The high ABR measurement threshold observed at the apex (8 kHz) without significant cell loss was likely to be due to high cellular stress conditions (Figure 1D) or limited hearing signal transmission due to SGN deformation and/or damage (Figure S1A,B).

The role of apoptosis in the development of hearing loss has been examined in many studies. The results of our in vivo and in vitro experiments demonstrated that auditory hair cell death is associated with ER stress-mediated UPR signaling, along with reduced HSF1 and HSP expression. This result is consistent with previous reports that intrinsic apoptosis is caused by Bax-dependent caspase-3 activity through CHOP under ER stress [37] (Figure 3 and Figure 4). In an HSF1 knockout model, hearing recovery was disturbed by noise overstimulation stress, which was considered similar to hair cell loss caused by ER stress [21]. Thus, HSF1 inhibits stress and apoptosis through HSP expression regulation and therefore plays an important role in hair cell survival.

Apoptosis related to the aging process is mainly caused by accumulated mitochondrial mutations, and mitochondrial-dependent apoptosis cannot be excluded from studies of aging animals [38,39]. However, intrinsic apoptosis also occurs due to Bax-dependent caspase activity, which was reduced by HSF1 overexpression, suppressing apoptosis (Figure 4). Therefore, in addition to mitochondrial damage, we conclude that ER stress is an important precursor of the intrinsic apoptosis pathway.

Previous studies have induced HSP expression through exposure to various types of stress such as heat, acoustic trauma, and ischemia or ototoxicity of the inner ear [40]. HSP70 is a potent cytoprotective HSP that was recently shown to be protective against ototoxic hair cell degeneration when overexpressed in cochlear and vestibular tissues through transgenesis [41,42]. Another study showed that an HSP70 viral vector overexpressed in non-sensory cells prevented hair cell death induced by utricle ototoxicity induced in vitro [43]. The interaction of a single HSP70 with multiple HSP40s generates unique HSP70–HSP40 pairs that facilitate specific processes at distinct locations within the cell; therefore, a mechanistic understanding of HSP40 function as a regulator of HSP70 is fundamental to understanding the cell biology of these molecular chaperones [44,45,46,47,48].

HSF1 responds to ER stress by controlling the refolding or degradation of misfolded or unfolded proteins, respectively, by increasing HSP levels. HSF1 transcription upregulates HSPs such as *Hspa1a* (HSP70) and *Dnajb1* (HSP40) [19]. In the present study, reduced levels of HSF1 and various HSPs were observed in premature senescence and in heat shock-exposed cell models and ARHL cochlear tissues (Figure 1E). Among these, the levels of HSP70 and HSP40 were significantly reduced; in cell experiments, HSF1 knockdown and overexpression dominantly regulated changes in their expression levels (Figure 4), and HSP70 and HSP40 expression were suppressed compared to the vehicle control upon the induction of heat shock after pretreatment with actinomycin D, a transcription inhibitor (data not shown). These results indicate that HSP70 and HSP40 were the main targets of HSF1 and that these co-chaperones play an important role in reducing ER stress. 

HSF1 regulates the expression of genes related to various pathways unrelated to HSPs [11,12,13,14]. In this study, we focused on the regulation of ER stress and HSPs by HSF1; however, HSF1 may also inhibit apoptosis by regulating other genes and pathways. A previous study reported that acute HSF1 depletion in human diploid fibroblast cells induces MDM2-p53-p21^WAF1/Cip1^-dependent cellular senescence through dehydrogenase/reductase 2 and DHRS2 [49]. In addition, suppression of HSF1 activates the p38–NF-κB-senescence-associated secretory phenotype (SASP) pathway, which in turn promotes DNA-damage-induced senescence. However, overexpression of HSF1 reduced senescence by inhibiting the p38–NFκB-SASP pathway [50]. These reports demonstrate another role of HSF1 in regulating cellular senescence. In the present study, DOXO induced DNA damage, inhibited the p53-p21^WAF1/Cip1^-dependent cell cycle and cell growth, and induced premature senescence. During these processes, several cells died whereas others induced senescence (Figure 2F). When premature senescence was induced, HSF1 activity decreased and that of p53-p21^WAF1/Cip1^ increased (Figure 2A,D). However, HSF1 overexpression resulted in a decrease in p53-p21^WAF1/Cip1^ activity (Figure 3E), inhibiting apoptosis (Figure 3D). This result suggests that HSF1 suppresses cellular senescence by reducing ER stress (Figure 3E) and regulating the cell cycle.

Our results suggest that decreased HSF1 expression may be a main cause of age-related hearing loss. However, the cause of the reduction in HSF1 protein levels during aging has not yet been identified. HSF1 degradation can be induced through CHOP activation via the UPR apoptotic pathway [51]. Furthermore, double phosphorylation of the HSF1 regulatory domain by ERK1 and GSK3 represses HSF1 via 14-3-3 recruitment and nuclear exclusion, which prevents HSF1 from binding to HSP gene promoters and repressing HSP transcription [52,53,54]. Thus, age-related changes in the levels and activities of ERK1, GSK3 and 14-3-3 may be involved in the progressive decline of the heat shock response. Additional diverse and complex mechanisms may also be involved; therefore, more research is needed.

A previous study used intratympanic delivery to penetrate the tympanic membrane and inject a water-soluble therapeutic agent into the middle ear, where it was taken up by oval or round window epithelial membranes. However, an amount of the reagent may have been lost to the Eustachian tube [55]. In this study, we diluted Lenti-viral *hsf1* or *vector* control with polybrene in water-soluble PBS, and then performed viral transduction via intratympanic delivery into the middle ear. After 3 months, we observed HSF1 expression at the base turn of the OC and partial expression at the middle and apex turns but could not clearly observe the effects on SGNs or the lateral wall (Figure S2). Our viral transduction results were consistent with our ABR results (Figure 5C). HSF1 expression was detected mainly at the base turn of the OC; we infer that most of the viral particles were taken up in this region, after which they migrated to the posterior vestibular artery and into general circulation via blood flow. However, further study is required to confirm this hypothesis. 

The first signs of age-related hearing loss (ARHL) appear in the high-frequency range, and hearing loss typically occurs within the high-frequency areas [34,35]. We found no significant inhibition of inner hair cell (IHC) and outer hair cell (OHC) loss in the basal turn region following HSF1 overexpression (Supplementary Figure S4A,B), while a hearing loss suppression effect was observed (Figure 5C). Cochlea tissue analysis confirmed that the gene expression and protein levels of HSF1, HSP70 and HSP40 increased, while cleaved caspase-3 and CHOP expression decreased (Figures S4C,D and 5D, respectively). Thus, inducing HSF1 expression during early-onset ARHL may alleviate hearing loss in the high-frequency region.

## 5. Conclusions

The results of this study suggest that the loss of auditory hair cells due to aging is involved in the mechanism of intrinsic apoptosis, and that ER stress is also involved in apoptosis mechanisms. We also confirmed that ER stress and apoptosis is regulated according to HSF1 expression levels; therefore, we cautiously suggest that it may be a useful therapeutic target for ARHL.

## Data Availability

Data available on request to the corresponding author.

## Supplementary Materials

The following are available online at https://www.mdpi.com/article/10.3390/cells10092454/s1, Figure S1: Histology of spiral ganglion neurons (SGNs) and immunofluorescence analysis of the organ of Corti (OC), SGNs, and SVs in the cochlear tissue of young and aged mice, Figure S2: Immunofluorescence analysis of the OC, SGNs, and lateral wall of age-related hearing loss (ARHL) cochlear tissues treated with vector or overexpressed HSF1, Figure S3: Time-course of HSF1 nuclear translocation and phosphorylation following heat-induced ER stress, Figure S4: IHC and OHC cell counts measured in histological sections from the apex to the basal turn in vector- and HSF1-treated mouse cochleae, Figures S5 and S6: The original film images of immunoblot analysis, Table S1: Real-time PCR primer list.
