# Supplementary material for: Heat Shock Factor 1 Prevents Age-Related Hearing Loss by Decreasing Endoplasmic Reticulum Stress
Source: Cells. 2021 Sep 17;10(9):2454. doi: 10.3390/cells10092454 (PMC8468389; doi:10.3390/cells10092454)
Supplement: Supplementary file 1 [file cells-10-02454-s001.zip › cells-1328627-supplementary.pdf]

Supplementary Table S1

Real-time PCR primer list

Target gene	Primer sequences
Hsf1-F	GGACAGGAACAGCTCCTTGA
Hsf1-R	TCCTGTTTCCCCTTCATCAG
Hspa1a(HSP70)-F	CAGCGAGGCTGACAAGAAGAA
Hspa1a(HSP70)-R	GGAGATGACCTCCTGGCACT
Dnajb1(HSP40)-F	CCCCATGCCATGTTTGCT
Dnajb1(HSP40)-R	GCGCTGCCCAAAAAAGG
18S rRNA -F	GTAACCCGTTGAACCCCATT
18S rRNA -R	CCATCCAATCGGTAGTAGCG

Supplementary Figure S1

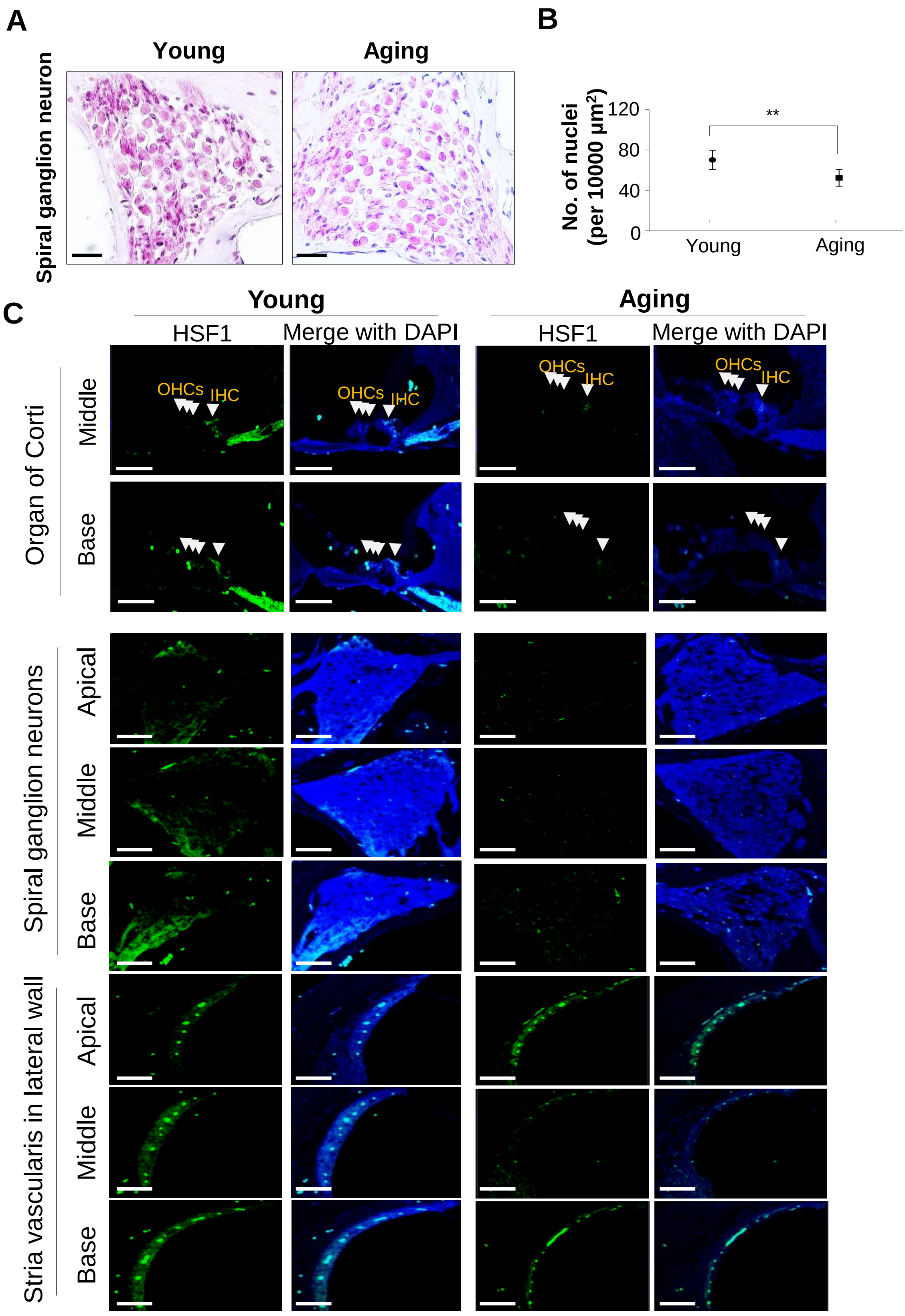

Supplementary Fgure S1. Histology of spiral ganglion neurons (SGNs) and immunofluorescence analysis of the organ of Corti (OC), SGNs, and SVs in the cochlear tissue of young and aged mice. **(A)** H & E-stained SGNs from the middle turn of the cochlea. Scale bars, 30 μm. **(B)** SGNs were quantified as the number of cell nuclei per 10,000 μm^2 in Rosenthal's canal at the middle and base turns. Data are means ± standard deviation (SD). $^{**}P < 0.01$ (Student's *t*-test). **(C)** Young and aging cochlea sections were immunolabeled with anti-HSF1 antibody. DAPI was used as a counterstain. White darts indicate inner (IHCs) and outer hair cells (OHCs). Scale bars, 30 μm.

Supplementary Fgure S2

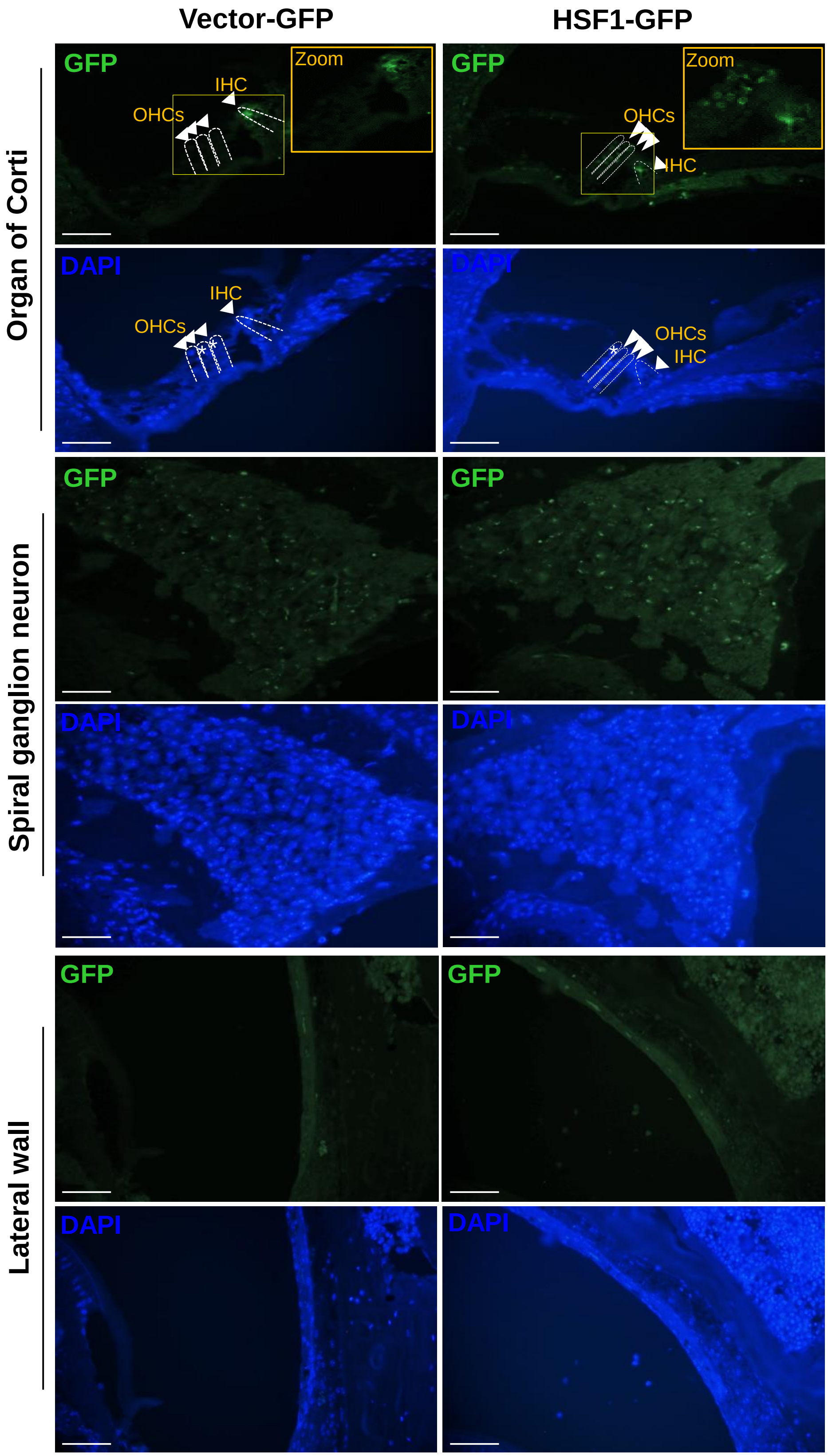

Supplementary Figure S2. Immunofluorescence analysis of the OC, SGNs, and lateral wall of age-related hearing loss (ARHL) cochlear tissues treated with *vector* or *hsf1*. Immunofluorescence of mGFP-tagged vector or HSF1 into OCs, SGNs, and the lateral wall were observed by confocal microscopy. Nuclei were stained with DAPI (blue). White darts indicate IHCs and OHCs within boxes. White asterisk indicates cell loss. Scale bars, 50 μm.

Supplementary Figure S3

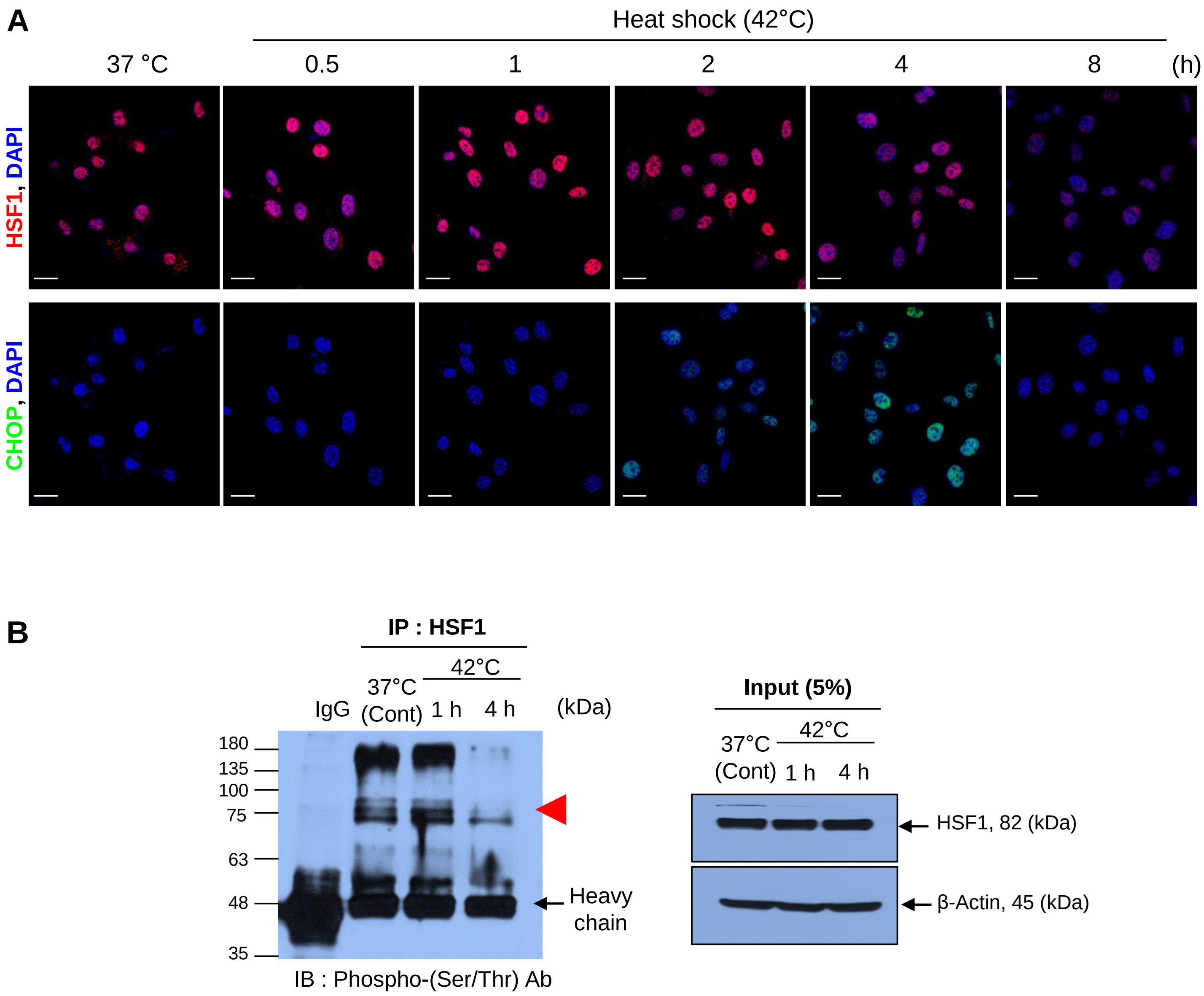

Supplementary Figure S3. Time-course of HSF1 nuclear translocation and phosphorylation following heat-induced ER stress. **(A)** Immunocytochemical analysis of HSF1 and CHOP (ER stress marker) using confocal microscopy. ER stress was induced at 42°C. Nuclei were stained with DAPI (blue). Scale bars, 20 μm. **(B)** Immunoprecipitation was performed using anti-HSF1 antibody. Phosphorylation of HSF1 was detected by phospho-(Ser/Thr) antibody. Red arrow heads indicate phosphorylation of HSF1. IgG heavy chain or input (5%) was used as the loading control.

Supplementary Figure S4

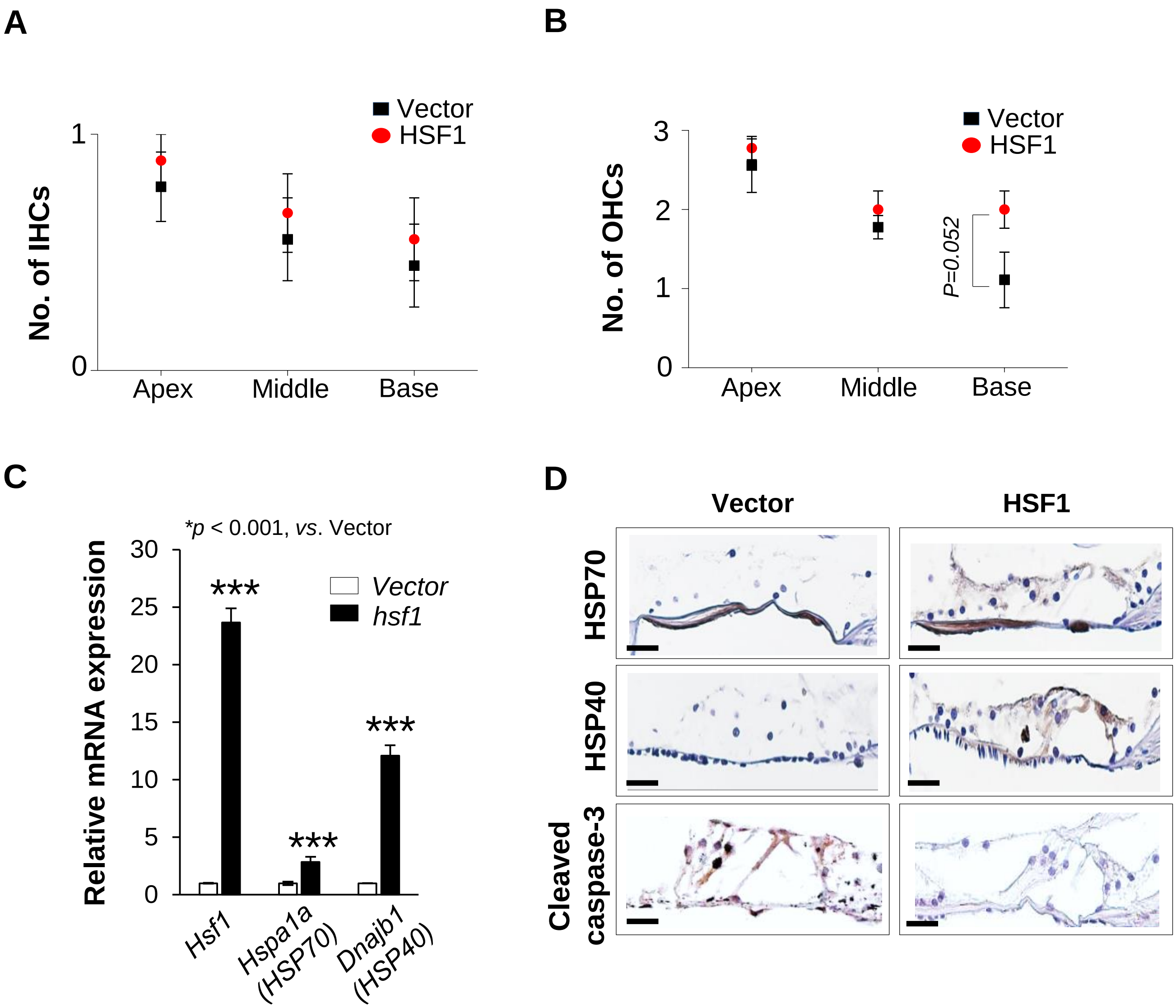

Supplementary Figure S4. (A) IHC and **(B)** OHC cell counts measured in histological sections from the apex to the basal turn in *vector*- and *hsf1*-treated mouse cochleae. Data are means ± standard error of the mean (SEM) of nine independent sections. $^{\#}P = 0.052$ [one-way analysis of variance (ANOVA), followed by Tukey's honest significant difference (HSD) test]. **(C)** *Hsf1*, *Hspa1a* (HSP70), and *Dnajb1* (HSP40) mRNA levels in *Vector*- and *hsf1*-treated mouse cochlear tissues. *Ribosomal RNA (18S)* was used as an internal control. Data are means ± SEM of three independent measurements. $^{***}P < 0.001$ (one-way ANOVA, followed by Tukey's HSD test). **(D)** *Vector*- and *hsf1*-treated cochlear sections were immunolabeled with anti-HSP70, anti-HSP40 and anti-cleaved caspase-3. Scale bars, 30 μm.

Supplementary Figure S5

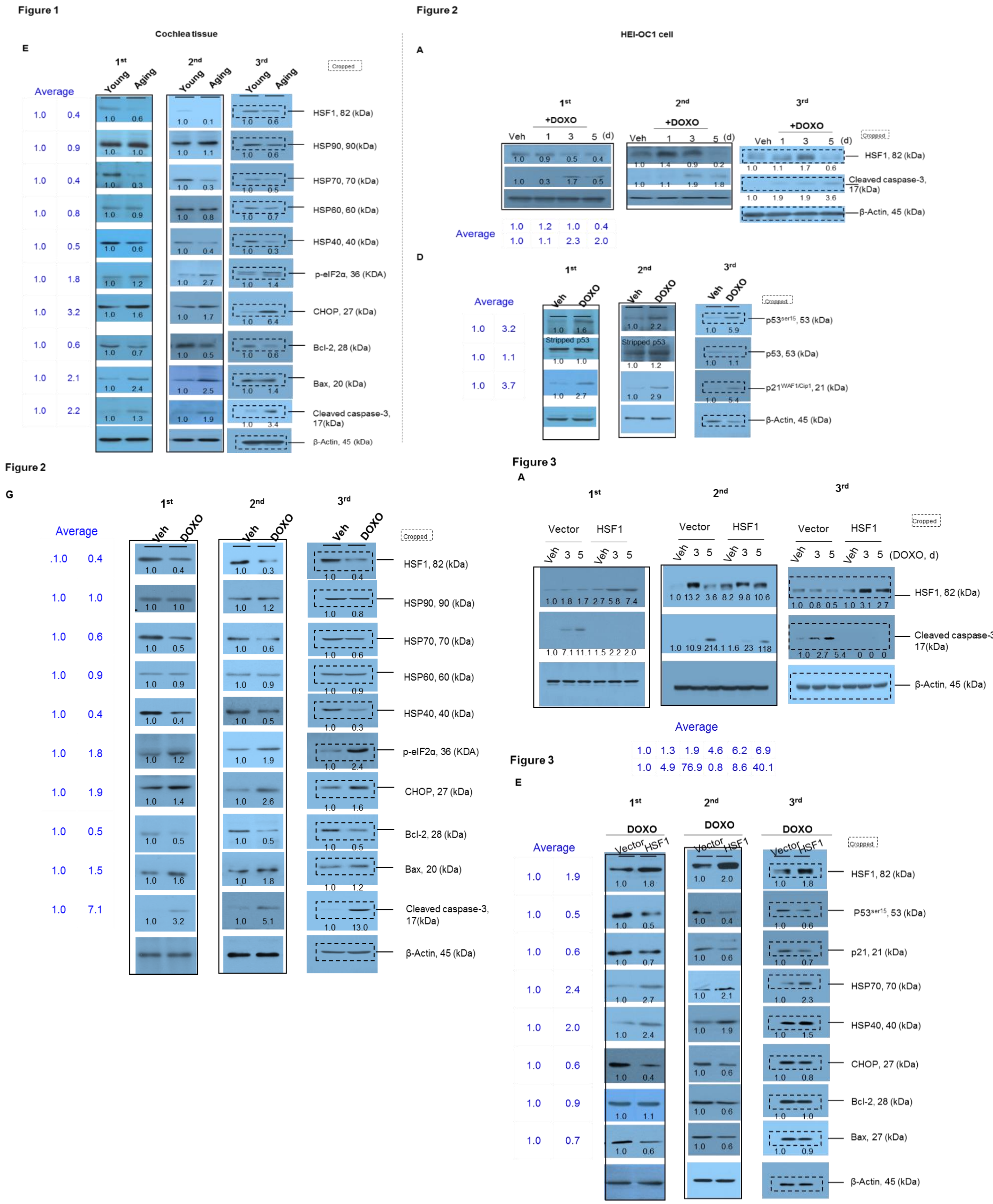

Supplementary Fgure S5. The original film images of immunoblot analysis. To minimize antibody loss and to allow synchronous detection of more than one protein on the same gel, membranes have been cut with a knife at the target protein molecular weights.

Supplementary Figure S6

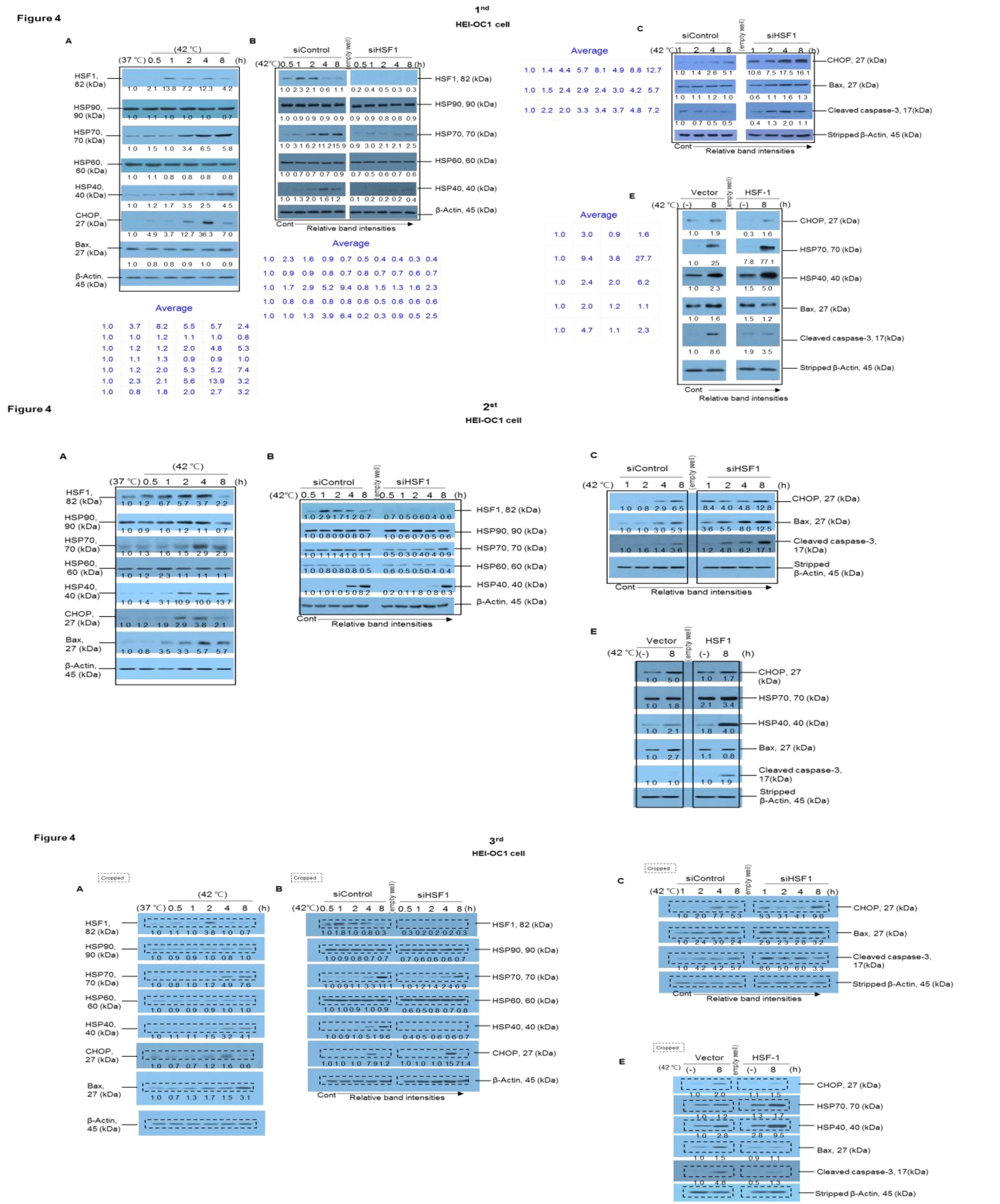

Supplementary Figure S6. The original film images of immunoblot analysis. To minimize antibody loss and to allow synchronous detection of more than one protein on the same gel, membranes have been cut with a knife at the target protein molecular weights.
